# Effects of OnabotulintoxinA on Habituation of Laser Evoked Responses in Chronic Migraine

**DOI:** 10.3390/toxins8060163

**Published:** 2016-05-25

**Authors:** Marina de Tommaso, Marianna Delussi, Katia Ricci, Anna Montemurno, Irene Carbone, Eleonora Vecchio

**Affiliations:** Neurophysiopathology of Pain, SMBNOS Department, Policlinico General Hospital, Giovanni XXIII Building, Via Amendola 207 A 70124 Bari, Italy; m.delussi@gmail.com (M.D.); katiari86@gmail.com (K.R.); annamonte@live.it (A.M.); ireneottavia.carbone@policlinico.ba.it (I.C.); eleonora.vecchio@uniba.it (E.V.)

**Keywords:** chronic migraine, neurotoxin mechanism, laser evoked potentials

## Abstract

Onabotulintoxin A (BontA) is an efficacious preventive treatment for chronic migraine, though the specific mechanism of action is still under discussion. The study aims: (1) To evaluate pain processing modifications in chronic migraine patients (CM) under single BontA administration in pericranial muscles, by means of CO^2^ Laser Evoked Potentials (LEPs) obtained by the stimulation of the skin over the right frontal and trapezius injection sites and hand dorsum, in a double blind placebo controlled crossover design. (2) To correlate main LEPs findings with clinical outcome after one year of BontA treatment. Twenty refractory CM patients were included in the analysis. The LEPs were recorded in basal conditions and seven days after BontA (PREEMPT protocol) and saline solution injection. The N1, N2 and P2 amplitude and latencies and N2P2 habituation index were evaluated and correlated with the percent change of headache frequency after one year of toxin treatment. After seven days of BontA treatment, a normalization of the trigeminal habituation index was observed, which was correlated with the clinical outcome after one year of BontA therapy. Patients displaying trigeminal LEPs facilitation at T0 time showed a more efficient therapeutic outcome. Neurotoxin may exert a modulating effect on trigeminal nociception, normalizing central neurotransmission.

## 1. Introduction

OnabotulintoxinA (BontA) is an efficacious preventive treatment for chronic migraine [1,2,3], though the specific mechanism of action is still under discussion [4,5]. Based on preclinical studies, the well-known action of BontA is the inhibition of the release of excitatory neurotransmitters from both motor and sensory neurons by preventing vesicle fusion to the cell membrane [6]. Apart from the inhibition of the release of acetylcholine, resulting in muscle paralysis, the internalization of the neurotoxin in sensory neurons that innervate the skin and muscles could potentially inhibit the release of pro-inflammatory mediators at several sites within the sensory neuron and suppress neurogenic inflammation near the injection site by preventing the release of the neuropeptides calcitonin gene-related peptide (CGRP) and substance P from free nerve endings [7,8]. In addition, the neurotoxin can exert a modulating effect on central sensitization by blocking the release of CGRP and glutamate from nociceptive nerve fibers terminating in the spinal cord, thus suppressing the stimulation of second-order neurons and glial cells [4,9,10,11]. The role of CGRP and glutamate in chronic migraine pathophysiology and related phenomena of central sensitization is supported by numerous evidences [12]. The observations about the low efficacy of BontA in episodic migraine compared to the good results in chronic forms [13] may suggest that the main action is exerted on the phenomena of central sensitization, which subtend the transition from sporadic to continuous headache, rather than toward factors initiating migraine attacks. Moreover, the inhibitory effects of BontA are also likely to involve the activity of myogenic trigger points, decreasing the persistent nociceptive barrage that promotes and maintains central sensitization [5]. The knowledge of mechanism of BontA in chronic migraine may improve its clinical employment and the individuation of patients with the best therapeutic response. Up to now, central sensitization changes induced by BontA were mainly evaluated by means of clinical questionnaires [14]. Neurophysiological methods may provide for an objective examination of nociceptive pain pathway functions and their modification in chronic pain syndromes. Laser evoked potentials (LEPs) are a useful tool for this purpose [15], and were applied in migraine to detect the abnormal elaboration of painful stimuli under repetitive stimulation and cognitive trials [16]. In particular, reduced habituation to repetitive multimodal stimuli is a well documented inter-critical feature of migraine, which involves pain pathways and may partly explain their persistent activation [17]. In addition, LEPs have been previously employed in episodic migraine to measure the effects of symptomatic and preventive treatments on pain pathways [18,19], while in medication overuse headache, the recovery of reduced habituation indicated a normalization of pain processing after detoxification [20].

In accord with previous studies [21] reporting that the inhibitory effect of the neurotoxin on nociceptive system after seven days of its injection could partly represent the mechanism of long-term clinical efficacy in chronic migraine, we aimed to:

(1) Evaluate LEPs changes at the main sites of injection, as frontal and trapezius sites, with a control site as the hand dorsum, after seven days of single BontA treatment, according to PREEMPT protocol [1], in a double blind placebo controlled crossover design (first outcome: LEPs modification after seven days of BontA injection)
(2) Correlate main LEPs findings with clinical outcome after one year of BontA treatment (second outcome: correlation between LEPs changes seven days after single BontA treatment with clinical improvement after one year (four treatment cycles) [1]).

## 2. Results

The LEPs performed at T0 and T1 before placebo or BontA were almost super-imposable, and statistical analysis of Student’s *t*-test did not report relevant differences, so we decided to consider the average values across the two recording series. All patients were recorded during or in close relationship with migraine occurrence (mean time from last migraine episode 6 ± 3.4 h; mean time from next migraine attack 5 ± 2.3 h). No patient was recorded during a crystal day.

The N1, N2 and P2 amplitudes and latencies in basal conditions were within normal limits for all the stimulation sites.

### 2.1. Aim 1

#### 2.1.1. Laser Pain Perception

**Figure 1:**
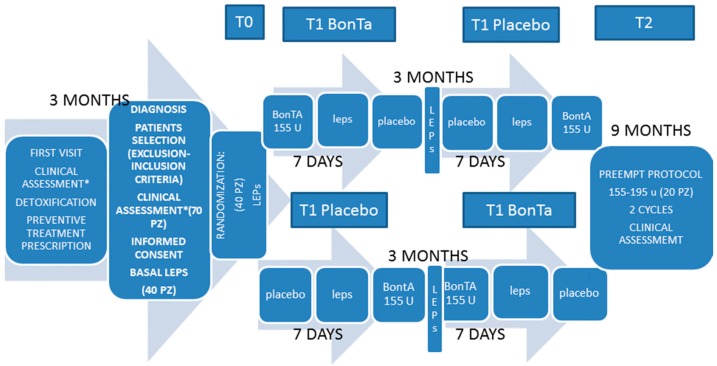
Main steps of experimental procedure are summarized. * Clinical assessment was based on frequency of headaches, as average number of headaches/day in one month, averaged over three months, allodynia questionnaire, Total Tenderness Score (TTS), Migraine Disability Assessment (MIDAS) score, headache intensity from 1 (slight) to 3 (maximum pain).

At T0 time, subjective pain induced by laser stimuli was significantly increased in CM patients compared to healthy controls at trigeminal level (controls trigeminal Visual Analogic Scale (VAS): 41.32 ± 12.2; CM patients 54.78 ± 21.84: ANOVA F 4.90 *p* = 0.02). The VAS patients’ values are reported in Table 1. At T1 time, BontA injection caused a slight and not significant laser pain perception decrease by the hand, trapezium and frontal levels, and also placebo exerted no relevant effect (Figure 1, Table 1).

#### 2.1.2. LEPs Latencies

At T0 time, latencies were within normal limits. In the T1 BontA condition, a significant prolongation of N2 and P2 latencies was found at the hand level, in respect to T0 and T1 placebo conditions, with a similar though not significant trend for N1 component. When the recordings from the skin over trapezius and frontal muscles were considered, no significant difference was found among the different conditions (Figure 1, Table 2).

#### 2.1.3. LEPs Amplitudes

At T0 time, LEPs amplitudes were not different from the control group. Moreover, the N1 and N2P2 amplitudes were similar between T0, T1 placebo and T1 BontA conditions (Figure 1, Table 3).

#### 2.1.4. N2-P2 Habituation

**Figure 2:**
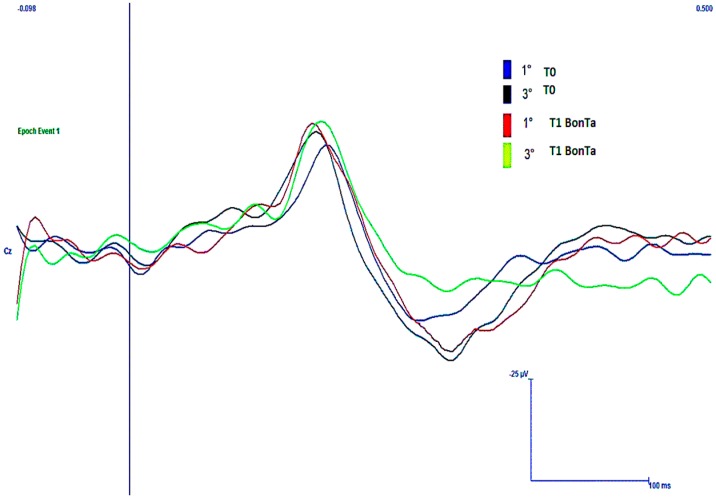
An example of N2P2 laser evoked responses by frontal stimulation in basal conditions (T0) and seven days after neurotoxin injection (T1 BontA), according to PREEPMT protocol [1] in a representative patient, who passed from a clear pattern of dis-habituation and facilitation to a more physiological habituation. 1°, first block of responses; 3°, third blocks of responses. Each trace is the average of seven artifact-free responses.

**Figure 3:**
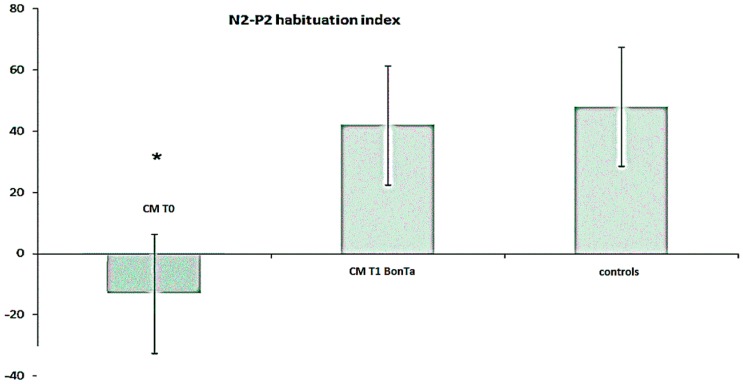
Trigeminal Laser Evoked Potemtials (LEPs) habituation index mean values and standard deviation in Chronic Migraine (CM) patients (*n* = 20) before T0 and seven days after BontA injection (T1 BontA), compared to controls (*n* = 20). The Bonferroni test showed that in CM group, trigeminal habituation was similar to controls after toxin treatment, while it was significantly reduced in basal conditions. Bonferroni test: T0 *vs.* T1 BontA and controls * *p* < 0.05.

At T0 time, the vertex complex showed reduced habituation in respect to control group when the face and the hand were considered, while a not significant trend was observed in the skin over the trapezius stimulation (for mean values in CM, see Table 3; in the controls, habituation index for face stimulation was 48.8% ± 22.2% ANOVA F 5.71, *p* = 0.011; hand stimulation 49.9 ± 27.7 F 4.68, *p* = 0.023; and trapezius 41.1 ± 21.2 F 3.19, *p* = 0.065). At trigeminal level, habituation index was significantly increased in T1 BontA condition, while at the hand and shoulder levels, there was a not significant trend toward a habituation index increase after toxin treatment (Table 3). In CM patients, at trigeminal level, BontA restored habituation within normal limits (Figure 2 and Figure 3). In addition, we observed that the effect of BontA of trigeminal habituation prevailed in patients with more evident LEPs abnormalities, as there was a significant correlation between the habituation index at T0 and percent degree of habituation changes induced by BontA in T1 condition (Pearson correlation 5.12, *p* = 0.01).

### 2.2. Aim 2: Clinical Outcome and Correlation with LEPs Findings

At T0 LEPs features, including trigeminal habituation, were not correlated with headache frequency, Migraine Disablity Assessment score (MIDAS), allodynia and Total Tenderness Score (TTS), though the negative correlation between allodynia and trigeminal habituation index approached the statistical significance (Pearson correlation = 0.4226, *p* = 0.051). We found that both allodynia and TTS influenced headache severity (Pearson correlation between allodynia and MIDAS: 0.569 *p* = 0.004; TTS and MIDAS 0.604 *p* = 0.001).

**Figure 4:**
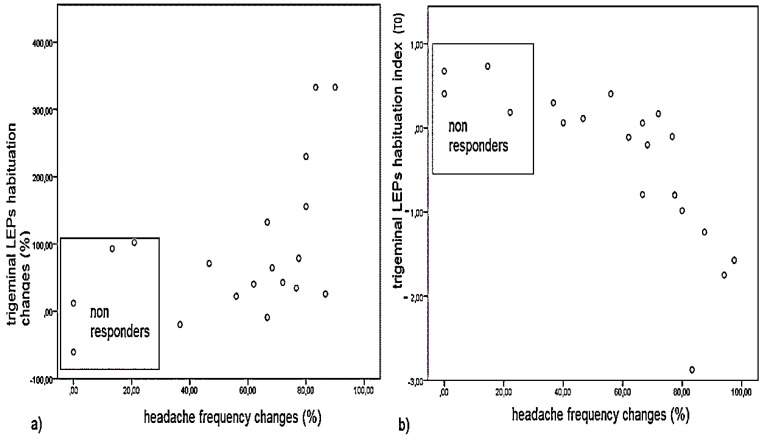
*X*-*Y* scatterplots to show correlations between trigeminal LEPs habituation at T0 and changes induced by toxin injection as well as clinical outcome after one year of BontA treatment: (**a**) Pearson correlation = 0.512, *p* = 0.011; and (**b**) Pearson correlation = 0.569, *p* = 0.004.

Headache frequency, MIDAS score and allodynia were significantly improved at T2 time (Table 4), while pericranial tenderness was only slightly reduced. Four patients reported high headache frequency and only a mild reduction of headache days at T2 time, so they were considered as non-responders. Interestingly, clinical outcome in terms of headache frequency reduction at T2 was significantly correlated with the effect of BontA on trigeminal LEPs habituation at T1 time and trigeminal habituation index recorded at T0 time, so the non-responders were characterized by higher trigeminal habituation index and a consequent reduced effect of toxin on this LEP pattern (Figure 4). Brachial LEPs latency changes were not correlated with modification of headache frequency induced by one year of toxin treatment (Pearson correlation 0.21, not significant—n.s.).

## 3. Discussion

The results of the study may be summarized as follows: after seven days of BontA treatment, there was no direct inhibitory effect on LEPs responses from the skin sites corresponding to the frontal and trapezius sites of injection, while a modulation of the trigeminal habituation index and its restoration into normal limits was observed. This pattern correlated with the clinical outcome after one year of BontA therapy. The more efficient was the therapeutic action, the more evident appeared the pattern of reduced trigeminal habituation at T0 time.

All the results will be commented on detail in the following sections.

### 3.1. Aim 1

#### 3.1.1. Main Effects of BontA on Subjective Laser Pain and LEPs Amplitude and Latency

BontA failed to exert any relevant effect on subjective laser pain seven days after injection. In line with laser pain perception, the LEPs amplitudes and latencies evoked by stimuli delivered over the sites of injection, remained quite similar after both BontA and placebo. The LEPs were previously employed to test the possible effect of symptomatic and preventive treatment on nociceptive pathways in episodic migraine. Both triptans and non-steroidal anti-inflammatory drugs reduced the vertex complex amplitude during a migraine attack, which effect was correlated with therapeutic efficacy, probably due to a direct action on trigeminal afferents and cortical sites elaborating the attention and emotional compounds of pain [18]. This evidence in humans is supported by animal experiments where nociceptive evoked response amplitude was sensitive to the pharmacological modulation of trigeminal activation [22,23]. Apart from the well-known direct action of BontA in attenuating nociceptive input from the muscles [24], it has also been proposed that, when injected peripherally, it can access the CNS, decreasing synaptic transmission in pain pathways [10]. It was supposed on the basis of several evidences that internalization of the neurotoxin in the sensory nerves that innervates the muscles and the skin, would result in a central effect by blocking CGRP and Glutamate in the spinal cord, thus inhibiting second order neuron transmission [5]. In this case, the neurotoxin intramuscular injection would have exerted a central inhibiting effect on nociceptive neurons receiving inputs from the skin topographically related to the muscular sites of inoculation, with a reduction of the central transmission of laser stimuli, which was not observed for LEPs evoked by frontal and shoulder stimulation. Moreover, we cannot exclude an analgesic effect on laser pain before and after the seventh day, though we choose this interval, in accord with the current knowledge about the time course of neurotoxin action [25]. We also failed to observe a significant placebo effect on subjective laser pain perception, despite the therapeutic procedure was complex enough to induce suggestion [26] and in previous studies LEPs were found to be sensitive to the placebo effect in migraine patients and healthy subjects [27,28]. Probably the slight muscles discomfort that patients complained in the sites of injection should have reduced the analgesic suggestion; in addition, the placebo effect may be reduced after seven days from the sham procedure. The slight but significant prolongation of vertex wave latencies for hand stimulation is really difficult to be explained, considering that we added the hand as a control site. We can suppose that seven days after BontA injection a weak inhibition of nociceptive transmission to the cortical zones generating the vertex waves [29] may occur only in the case of somatic but not trigemino-cervical stimuli, which cortical processing is facilitated in the acute phase of migraine [30]. Moreover, in our previous studies trigeminal facilitation was never expressed by clear latency reduction, so this result remains partially unexplained and worthy of confirmation by case series enlargement and observation time extending. In any case, the lack of effect of BontA on LEPs evoked by the stimulation of the skin over the sites of injection, seems to be in contrast with a direct inhibitory effect of the neurotoxin on the trigeminal and the cervical nociceptive afferent system, at least seven days after treatment.

#### 3.1.2. Effects of BontA on LEPs Habituation

At T0 time our CM patients showed a tendency toward a progressive vertex complex increase instead of reduction at trigeminal level, which pattern could represent the electrophysiological counterpart of central sensitization of cerebral structures subtending pain processing, probably related to the mechanism of migraine attack and evolution into chronic form [31,32,33,34]. In accord with this hypothesis, our patients showed facilitation instead of habituation of trigeminal LEPs, given that the majority of them were recorded during or in close temporal relationship with migraine, or in any case during non-migraine headache. The trigeminal LEPs habituation was found particularly compromised in our patients, though the phenomenon of reduced habituation interested also the LEPs recorded from the hand dorsum, in accord with previous studies [31] and evidence about the spreading of central sensitization at the somatic level [35]. Though the trigeminal habitual pattern was clearly different from normal controls, it was not significantly correlated with headache severity, which was similar across refractory migraine patients.

Toxin injection showed a clear effect on trigeminal LEPs habituation, which was restored within normal limits, changing from a pattern of slight facilitation to a more physiological pattern of progressive vertex complex amplitude reduction. The effect of the neurotoxin was proportional to the severity of basal habituation impairment, being more effective in patients showing facilitation of trigeminal LEPs. The mechanisms by which neurotoxin could induce a modulation and normalization of trigeminal habituation pattern, may be based on the inhibition of muscle nociceptors, with a normalization of the afferent firing toward trigeminal second-order neurons, receiving inputs from the meninges and vascular structures, responsible for migraine pain. Alternatively, the direct effect on caudal trigeminal nucleus would induce an increase of the inhibitory controls on neuro-transmission, limiting the excess of incoming painful inputs from the trigeminal nociceptors in those patients displaying facilitation instead of habituation of pain stimuli processing. Thus, while a direct neurotransmission block would induce an amplitude reduction of averaged LEPs [36], a modulation of first-second order nociceptor synapses may result in a normalization of abnormal firing of painful inputs through the cortex. Moreover, there was no correspondence between LEPs reduced habituation pattern and LEPs amplitude, which may be explained by the variability of the amplitude of the first LEPs block and the degree of N2P2 facilitation *vs.* reduction in the course of repetitive stimulation, factors influenced by the temporal proximity of migraine attacks and the effect of preventive and symptomatic treatments [19,20,30,31,32,33]. In previous studies, preventive treatment with topiramate, was also able to modulate the N1 habituation in episodic migraine, with a significant correlation between clinical effects and the normalization of neurophysiological responses [19]. In our study, we decided to analyze only habituation of vertex N2-P2, considering the small amplitude of N1 [15] and the possible further reducing effect caused by symptomatic treatments, which were not discontinued during the observation period. Reduced habituation interested in a slight and not significant way also the LEPs obtained from the skin over the trapezius, corresponding to the superior cervical system, which is in strict functional relationship with the trigeminal caudal nucleus and involved in the migraine central sensitization phenomena [35]. The lack of significance of this pattern with respect to control values could be due to a variety of expression and diffusion of central sensitization and reduced habituation pattern across patients, so this pattern would deserve further confirmation in larger series. Summarizing, BontA seemed to modulate trigeminal habituation in those patients where abnormality of this pattern was more evident, with no effect at trapezius level, where N2-P2 habituation was not clearly modified in respect to controls. The lack of normalizing effect at the hand level may be caused by the distant site of BontA injection from the site of laser stimulation and the less evident pattern of reduced habituation with limited boundaries for BontA action.

### 3.2. Aim 2: Correlation between Effect of Neurotoxin on LEPs Features and Long-Term Clinical Outcome

Our CM patients showed a favorable outcome of their headache frequency and severity at T1 time after one-year follow-up, according to previous studies conducted in large migraine populations [37]. Their improvement consisted of headache days and migraine intensity reduction, limited disability and fewer allodynia symptoms, in accord with previous reports [14]. Our patients were not affected by Medication Overuse Headache (MOH) [38], as we tried preventively to discontinue current symptomatic treatment without success on migraine improvement, so we did not consider the number of analgesics intakes in the clinical outcome, as its reduction was the consequence of CM improvement. Moreover, new evidences are available about the efficacy of BonTa on CM with associated MOH, so in our patients a beneficial effect due to the reduction of analgesic use could not be excluded [38]. At T0 time, central sensitization symptoms influenced the severity of migraine, as shown by the positive correlation between allodynia, pericranial tenderness and MIDAS scores. While allodynia was reduced after four treatment cycles, pericranial tenderness showed a limited improvement, that could suggest only a slight long term effect on muscle nociceptors and the possibility that clinical efficacy is not mediated by pericranial muscle relaxation. The central modulating effect on trigeminal neurotransmission, may explain both the short-term normalization of LEPs habituation and the long-term clinical improvement with allodynia symptom reduction, though a mild effect on muscle nociceptors could not be excluded. In our small CM group, four patients reported a persistent headache frequency >15 days/month and <50% reduction of headache days at T1 time. The case number was limited to characterize the clinical feature of non-responders, but we can try to suggest that the neurophysiological pattern of habituation of trigeminal LEPs, instead of facilitation, could delineate the toxin resistant migraine phenotype. We can in fact advance this risky hypothesis, basing on the positive correlation between the degree of trigeminal LEPs habituation and clinical improvement, as far as patients with less abnormalities in trigeminal habituation and probably sensitization, are not influenced by the modulating effect on central pain transmission exerted by the neurotoxin. Other factors beyond trigeminal central sensitization may cause evolution into chronic migraine in these patients, though at this stage we cannot establish which clinical feature may explain their toxin resistant profile, as well as their trigeminal habituation pattern. In basal conditions, trigeminal LEPs habituation did not correlate with any clinical feature, including allodynia symptoms, though the correlation approached statistical significance. The enlargement of case series, would help us to understand if the trigeminal LEPs habituation index may be a sign of the severity of central sensitization, probably normalized by the neurotoxin, or if other factors, for example psychiatric features, may be the main causes of chronic migraine.

### 3.3. Study Limitations

Many problems may constitute a limitation for the significance of our result. The small case series reduced the reliability of results, though the neurophysiological procedures request time and collaboration, which is difficultly obtained by patients who complain invalidating migraine. The low compliance in clinical diaries also caused the exclusion of many patients, though we were very rigorous on this matter, in order to report clinical outcome in a reliable way. The placebo-controlled study design caused half patients to be recorded after three months from the first toxin cycle, though neurophysiological pattern was similar to the basal condition for the long time elapsing from the injection. Patients were under symptomatic and preventive therapies, giving that our study was observational under the clinical point of view, so we were not allowed to discontinue treatments. Finally, the seven days after effects of BontA on LEPs could not completely explain the long-term efficacy mechanism in chronic migraine.

## 4. Conclusions

The results of this neurophysiological double blind, placebo controlled trial on main effects of BontA treatment on CM may suggest that the neurotoxin could exert a modulating effect on trigeminal nociception and altered pattern of habituation, normalizing central neurotransmission. Normalization of LEP habituation seven days after BontA injection therapy in CM patient characterized by a pattern of trigeminal LEP facilitation may have predictive value for long-term therapeutic outcome. The challenge for future studies would be the enlargement of case series, and the individuation of clinical phenotype corresponding to the neurophysiological pattern predictive of good therapeutic response.

## 5. Materials and Methods

### 5.1. Patients

Cases selection was made from September 2013 to February 2014. Outpatients attending for the first time the tertiary headache Center named “Headache Ambulatory of Neurophysiopathology of Pain Unit of Bari Aldo Moro, University”, were considered for study selection after three months observation, during which they were requested to fill a headache diary and the allodynia questionnaire [39]. Chronic Migraine diagnosis was made, according to the last classification [37]. Patients with probable chronic migraine emerging from the first visit were initially assigned to preventive treatment, depending upon their clinical and pharmacological history, and in accordance with Italian guidelines [40]. Patients with medication over-use, were submitted to detoxification by means of corticosteroids, and assigned to preventive treatment. After three months of observation, patients with refractory Chronic Migraine [41] (as requested by the Italian Government rules on the public access to Neurotoxin treatment) were assigned to BontA protocol, in accord with PREEMPT study design [1]. Exclusion criteria for the study were: concomitant neurological, general medical or psychiatric diseases and a not complete compliance in clinical diaries compilation. All preventive and symptomatic therapies were left unmodified during the neurophysiological study and the clinical observation. 

After the first visit, a total of 70 patients was selected for BontA treatment, 40 patients gave the written informed consent before they participated in the study. Twenty patients completed the study, while the others were excluded, 12 for a not complete compliance in diaries completions, and the remainder for not reliable LEPs recordings, most of these for a scarce compliance due to the concomitance of severe migraine and associated symptoms. Eleven excluded patients had been firstly randomized to placebo, the remainder to BontA. Demographic and clinical features of CM patients are summarized in Table 4. All patients were originating and resident in Southern Italy. The LEPs from 20 controls comparable for sex (18 females and 2 males), age (44.9 + 12.1), origin and permanent address on the basis of the absence of neurological, psychiatric, general medical diseases and first degree inheritance for migraine, were also compared with those of patients included. They were volunteers, selected among the Hospital and University staff.

### 5.2. Study Design

This was a double blind crossover randomized, placebo controlled study on effects of BontA on LEPs (Figure 1), completed by a correlation of main LEPs findings with clinical outcome after one year of treatment. Patients and medical and technical staff were in blind conditions during toxin or placebo injection, clinical and neurophysiological examination and LEPs analysis. Toxin and placebo injection were prepared by an expert nurse, who assigned a code to patients and experimental sessions. The study was approved on 1 July 2013 by the local ethical committee of the Bari Policlinico General Hospital (identification code: 1289), and was entirely conducted in accord with the Declaration of Helsinki of 1975 (http://www.wma.net/en/30publications/10policies/b3/), revised in 2008. Patients signed a written informed consent on a placebo controlled study, limited to neurophysiological examination. After the first visit, a three-month observation was carried out (Figure 1). At this time, patients fulfilling the inclusion/exclusion criteria and assigned to onabotulintoxinA treatment [1] were requested to participate in the neurophysiological study. At the T0 time (Figure 1), patients were randomly assigned to placebo or BontA injection, and LEPs were recorded after seven days (T1 time, Figure 1). We choose this interval, in accord with the current knowledge about the time course of neurotoxin action [25]. Patients assigned to placebo were treated by BontA at this time, and the inverse occurred in toxin group. The placebo treatment consisted of intramuscular injections of a minimum (0.1 cc) of saline solution in 31 pericranial points near those injected with BontA. Patients were advised that the placebo procedure served for the neurophysiological examination, and that the real treatment would be done in any case for therapeutic purpose. Considering the high migraine frequency characterizing our patients, it would be impossible to obtain LEPs during inter-critical phases, so we decided to schedule the recording dates independently from migraine occurrence, checking for headache diaries to establish the interval from the last and the next migraine attack or headache. Three months after the first session, a crossover with placebo or BontA was done, after which LEPs were again recorded as reported above (Figure 1). All patients were then requested to complete two further BontA cycles over a total observational time of one year, in accord with PREEMPT protocol [1]. The final clinical evaluation was carried out after three months of the fourth therapeutic session (T2 time, Figure 1).

### 5.3. Clinical Assessment

The frequency of headache and allodynia were assessed by the diaries reporting the number of days with headache and its intensity, in a score from 1 (slight) to 3 (maximum intensity), and allodynia questionnaires, which patients were requested to fill for any migraine episode [39]. The MIDAS scale was also employed to evaluate headache-related disability [42] and pericranial tenderness was also detected [43,44].

### 5.4. Aim 1

#### 5.4.1. Laser Evoked Potentials Examination−Stimulation Procedure

The pain stimulus consisted of laser pulses (wavelength 7.6 lm) that were generated by a CO^2^ laser (Neurolas Electronic Engineering, Florence, Italy). The stimulation site was visualized using a He–Ne laser beam. After each stimulation, the laser beam was slightly shifted to a nearby site to avoid nociceptor sensitization and skin damage.

The diameter of the laser beam was 2.5 mm, and the duration of the stimulus pulse was 30 ms. To define the pain threshold, single stimulus pulses were presented in a random order at 4–5 different intensities at 1.5 W intervals. The subjects were requested to report the quality of sensation and the pain threshold, which was expressed as the laser intensity (expressed in Watts) that produced a pinprick sensation followed by a burning sensation. Thirty consecutive laser stimuli were then delivered to any stimulation site at an intensity level set one step (1.5 W) above the pain threshold at an inter-stimulus interval of 7 s. We evaluated LEP habituation within single trials, to minimize exam duration and distress. The dorsum of the right hand, the right supraorbital zone and the skin over the right trapezius were stimulated. The order of site stimulation was randomized across sessions. The patients were requested to rate the laser pain intensity at the end of the stimulation series, according to a 0–100 visual analogical scale in which 100 represented maximal pain, shown in intense red color.

#### 5.4.2. Recording Procedure

Each subject was seated in a comfortable position in a quiet room with an ambient temperature of 21–23 °C in an awake and relaxed state with eyes open. All subjects and observers wore protective goggles during data acquisition. In addition to the 19 standard positions of the international 10–20 system, 37 electrodes were placed on the *x*, *y*, and *z* coordinates provided by Advanced Source Analysis (ASA) software (ASA version 4.7; ANT Software, Enschede, The Netherlands, 2014 http://www.antneuro.com). The reference electrode was placed on the nose, the ground electrode was in Fpz, and 1 electrode was placed above the right eyebrow for electro-oculographic (EOG) recording. The impedance was maintained at 7 kΩ or less. The EEG and EOG signals were amplified using a bandpass filter of 0.5–80 Hz, digitized at 250 Hz, and stored on a biopotential analyzer (Micromed System Plus, Micromed, Mogliano Veneto, Italy; www.micromed-it.com).

In the present study, we examined the waves obtained from the CZ position for the 10–20 International System (impedance below 5000 Ω), referred to the nasion, and those obtained from T3 derivation, referred to the Fz position, with the ground electrode at Fpz.

#### 5.4.3. Laser-Evoked Potential Analysis

An investigator who was blinded to the treatment analyzed the LEP recordings of 1 s, including 100 ms of pre-stimulus time, at a sampling rate of 256 Hz. All LEP recordings containing transient signals that exceeded 65 mV in any channel were excluded from the average by an automatic artifact rejection algorithm. Other artifacts were visually inspected. For each stimulation site, we evaluated the averages of at least 21 valid (artifact-free) responses, divided in blocks of at least seven artifact-free responses. The N1 component was analyzed at T3-Fz, and the N2 and P2 components were analyzed at the vertex (CZ-nasion) [15]. The absolute latencies of the scalp potentials were measured at the highest peak of each response component. The amplitude of each wave was measured from the baseline, in an automatic way of calculating the average signal on the entire sweep and subtracting this average signal from the trace (ASA version 4.7 by ANT software; Advanced Neuro Technology, Enschede, The Netherlands, 2014). The peak-to-peak amplitude was taken into consideration for the vertex biphasic LEP component (N2-P2). To assess N2-P2 habituation, we decided to consider only the initial and final block of single responses, considering that in migraine the trend of LEPs amplitude changes is quite irregular in the course of the stimulation, while reduced habituation is expressed by the lack of significant amplitude modification between the first and the last responses [45]. We simplified the habituation pattern computing the percent difference between the LEP amplitudes obtained in the first and third blocks of the averaged responses relative to the first block (first response-third response × 100/first response). This value was defined as the habituation index (HI). Positive values corresponded to a reduction of the N2-P2 amplitudes from the first to the third stimulation series, negative values expressed N2-P2 facilitation [46].

### 5.5. Statistical Analysis

We preliminary evaluated a sample size of minimum 20 patients for the first outcome, considering the single LEPs variables, with an expected 30% effect of placebo [17] and 60% effect of real treatment, with 20% of SD for a significance level of 5% and a power of 90%. For the second outcome, we considered proceeding with correlation, even though our sample size was far from the optimal one for reliable measures, to indicate a preliminary trend and directions for future studies.

The repeated measures ANOVA was carried out taking the VAS values, the N1 and N2P2 amplitude and the N1, N2 and P2 latencies as variables and the condition (a) basal *vs.* (b) seven days after BontA and (c) seven days after placebo as factors. A *post-hoc* Bonferroni test was also employed between the different conditions for single variables.

Clinical variables at T0 and T2 [1] were compared by means of Student’s *t*-test for paired data. The clinical scores obtained in the three months preceding the randomization and following the fourth treatment cycle were considered. The *p*-values used for statistical significance in this analysis was a one-sided alpha of 0.05, without adjustment for multiplicity. We considered as non-responder patients with a persistent headache frequency >15 days/month and <50% reduction of headache days in the last clinical control.

For correlations between LEPs and clinical features, the Pearson correlation test was carried out.

## Figures and Tables

**Table 1:** Mean values ± SD of 0–100 Visual Analogic Scale (VAS) values by laser stimuli. Results of ANOVA for repeated measures are reported. For experimental design, please refer to Figure 1.

Site	T0	T1 BontA	T1 Placebo
Hand	47.43	44.61	48.39
	17.13	20.53	19.31
ANOVA	-	-	-
F 0.66	-	-	-
P 0.53	-	-	-
Face	54.78	50.22	55.08
	21.84	17.99	19.53
ANOVA	-	-	-
F 1.76	-	-	-
P 0.2	-	-	-
Trapezius	54.45	51.41	55.32
	16.04	14.55	19.71
ANOVA	-	-	-
F 0.83	-	-	-
P 0.83	-	-	-

**Table 2:** Mean values ± SD of Laser Evoked Potentials (LEPs) latencies in the different conditions. Results of ANOVA for repeated measures and Bonferroni test are reported. The significant results are in bold. For experimental design, please refer to Figure 1.

Site	N1 T0	N1 T1 BontA	N1 T1 Placebo	N2 T0	N2 T1 BontA	N2 T1 Placebo	P2 T0	P2 T1 BontA	P2 T1 Placebo
Hand (msec)	159.11	169.54	159.23	206.1.2	228.45	200.18	339.34	362.45	325.29
	21.8	26.4	29.23	27.6	26.5	25.5	9.41	10.18	9.9
ANOVA	F 0.83	-	-	**F 4.97**	-	-	**F 3.94**	-	-
	P 0.45	-	-	**P 0.019**	-	-	**P 0.038**	-	-
	-	-	-	Bonferroni T1 BontA *vs* T0 and T1 placebo *p* < 0.05	-	-	Bonferroni T1 BontA *vs* T0 and T1 placebo *p* < 0.05	-	-
Face (msec)	135.23	137.43	138.96	185.34	187.18	190.38	304.44	307.14	309.41
	32.12	29.9	32.12	35.5	15.5	24.7	29.1	30.2	45.3
ANOVA	F 0.25	-	-	F 0.69	-	-	F 0.1	-	-
	P 0.78	-	-	P 0.37	-	-	P 0.9	-	-
Trapezius (msec)	155.11	160.23	156.43	202.34	203.21	208.37	335.34	344.23	337.16
	22.3	25.7	15.8	32.12	37.45	30.17	32.4	36.2	41.1
ANOVA	F 0.39	-	-	F 0.20	-	-	F 0.33	-	-
	P 0.68	-	-	P 0.82	-	-	P 0.72	-	-

**Table 3:** Mean values ± SD of LEPs amplitudes and habituation index of vertex complex in the different conditions. Results of ANOVA for repeated measures and Bonferroni test are reported. No significant result was found for amplitudes, while the significant increment of habituation index is in bold. For experimental design, please refer to Figure 1.

Site	N1 T0 (uV)	N1 T1 BontA (uV)	N1 T1 Placebo (uV)	N2-P2 T0 (uV)	N2-P2 T1 BontA (uV)	N2-P2 T1 Placebo (uV)	HAB-n2p2 T0%	HAB-n2p2 T1 BontA%	HAB-n2p2 T1 Placebo%
Hand	5.5	4.1	4.5	13.86	15.32	13.42	1.60	18.1	8.2
	3.9	4	2.9	9.92	11.25	11.39	-	-	-
ANOVA	F 0.23	-	-	F 0.32	-	-	F 132	-	-
	P 0.79	-	-	P 0.72	-	-	P 0.29	-	-
Face	7.26	8.28	6.91	16	22.77	20.13	−13.12	42.1	−2.81
	5.1	4.2	4.5	12.21	18.12	15.13	6.7	23.3	4.5
ANOVA	F 0.63	-	-	F 0.91	-	-	**F 5.58**	-	-
	P 0.54	-	-	P 0.43	-	-	**P 0.03**	-	-
	-	-	-	-	-	-	Bonferroni T1 BontA *vs*. T0 *p* < 0.002 T1 BontA *vs*. T1 placebo *p* < 0.01	-	-
Trapezius	4.88	6.32	5.79	13.12	15.13	13.67	16.4	12.8	14.8
	4.1	5.5	3.9	9.12	9.8	7.8	11.5	12.1	12.1
ANOVA	F 0.65	-	-	F 0.23	-	-	F 0.40	-	-
	P 0.53	-	-	P 0.69	-	-	P 0.67	-	-

**Table 4:** Demographic and clinical features of Chronic Migraine (CM patients). The results of Student’s *t*-test for paired data are reported. Significant results are in bold.

Chronic Migraine Patients N° 20	Age	Sex	Preventive Concomitant Treatments	Headache Frequency	Allodynia	Total Tenderness Score	MIDAS	Headache Intensity
basal	45.45 ± 11.7	18 f 2 m	10 topiramate (75–100 mg/die) 6 amitriptiline 10–25 mg/die 4 sodium vaproate (500–1000/die)	25.7 ± 3.7	3.65 ± 2.83	17.5 ± 4.94	79.4 ± 25.5	2.9 ± 0.14
one year observation (BontA treatment)	-	-	-	10.5 ± 7.3	2.25 ± 1.97	15.5 ± 8.7	29.55 ± 30.3	1.2 ± 0.5
*t*-test	-	-	-	7.97	3.39	1.72	3.85	7.96
*p*	-	-	-	**0.0001**	**0.003**	0.1	**0.001**	**0.0001**

## Abbreviations

The following abbreviations are used in this manuscript:

LEPS	Laser Evoked Potentials
CM	Chronic Migraine
TTS	Total Tenderness Score
VAS	Visual Analogic Scale
BontA	Onabotulintoxin A

## References

[1] Diener H.C., Dodick D.W., Aurora S.K., Turkel C.C., DeGryse R.E., Lipton R.B., Silberstein S.D., Brin M.F., PREEMPT 2 Chronic Migraine Study Group (2010). OnabotulinumtoxinA for treatment of chronic migraine: Results from the double-blind, randomized, placebo-controlled phase of the PREEMPT 2 trial. Cephalalgia.

[2] Dodick D.W., Turkel C.C., DeGryse R., Aurora S.K., Silberstein S.D., Lipton R.B., Diener H.C., Brin M.F., PREEMPT Chronic Migraine Study Group (2010). OnabotulinumtoxinA for treatment of chronic migraine: Pooled results from the double-blind, randomized, placebo-controlled phases of the PREEMPT clinical program. Headache.

[3] Aurora S.K., Winner P., Freeman M.C., Spierings E.L., Heiring J.O., DeGryse R.E., VanDenburgh A.M., Nolan M.E., Turkel C.C. (2011). OnabotulinumtoxinA for treatment of chronic migraine: Pooled analyses of the 56-week PREEMPT clinical program. Headache.

[4] Matak I., Lacković Z. (2014). Botulinum toxin A, brain and pain. Prog. Neurobiol..

[5] Durham P.L., Cady R. (2011). Insights into the Mechanism of OnabotulinumtoxinA in Chronic Migraine. Headache.

[6] Humeau Y., Doussau F., Grant N.J., Poulain B. (2000). How botulinum and tetanus neurotoxins block neurotransmitter release. Biochimie.

[7] Aoki K.R. (2003). Evidence for antinociceptive activity of botulinum toxin type A in pain management. Headache.

[8] Dolly O. (2003). Synaptic transmission: Inhibition of neurotransmitter release by botulinum toxins. Headache.

[9] Simpson L. (2013). The life history of a botulinum toxin molecule. Toxicon.

[10] Cairns B.E., Gazerani P. (2014). Botulinum neurotoxin A for chronic migraine headaches: Does it work and how?. Pain Manag..

[11] Cernuda-Morollón E., Ramón C., Martínez-Camblor P., Serrano-Pertierra E., Larrosa D., Pascual J. (2015). OnabotulinumtoxinA decreases interictal CGRP plasma levels in patients with chronic migraine. Pain.

[12] Van Dongen R.M., Zielman R., Noga M., Dekkers O.M., Hankemeier T., van den Maagdenberg A.M., Terwindt G.M., Ferrari M.D. (2016). Migraine biomarkers in cerebrospinal fluid: A systematic review and meta-analysis. Cephalalgia.

[13] Aurora S.K., Gawel M., Brandes J.L., Pokta S., Vandenburgh A.M. (2007). Botulinum toxin type A prophylactic treatment of episodic migraine: A randomized, double-blind, placebo-controlled exploratory study. Headache.

[14] Hollanda L., Monteiro L., Melo A. (2014). Botulinum toxin type a for cephalic cutaneous allodynia in chronic migraine: A randomized, double-blinded, placebo-controlled trial. Neurol. Int..

[15] Treede R.D., Lorenz J., Baumgärtner U. (2003). Clinical usefulness of laser-evoked potentials. Neurophysiol. Clin..

[16] De Tommaso M. (2008). Laser-evoked potentials in primary headaches and cranial neuralgias. Expert Rev. Neurother..

[17] De Tommaso M., Ambrosini A., Brighina F., Coppola G., Perrotta A., Pierelli F., Sandrini G., Valeriani M., Marinazzo D., Stramaglia S. (2014). Altered processing of sensory stimuli in patients with migraine. Nat. Rev. Neurol..

[18] De Tommaso M., Losito L., Libro G., Guido M., di Fruscolo O., Sardaro M., Sciruicchio V., Lamberti P., Livrea P. (2005). Effects of symptomatic treatments on cutaneous hyperalgesia and laser evoked potentials during migraine attack. Cephalalgia.

[19] Di Clemente L., Puledda F., Biasiotta A., Viganò A., Vicenzini E., Truini A., Gruccu G., di Piero V. (2013). Topiramate modulates habituation in migraine: Evidences from nociceptive responses elicited by laser evoked potentials. J. Headache Pain.

[20] Ferraro D., Vollono C., Miliucci R., Virdis D., de Armas L., Pazzaglia C., Le Pera D., Tarantino S., Balestri M., di Trapani G. (2012). Habituation to pain in ‘’medication overuse headache’’: A CO_2_ laser-evoked potential study. Headache.

[21] Gazerani P., Staahl C., Drewes A.M., Arendt-Nielsen L. (2006). The effects of Botulinum Toxin type A on capsaicin-evoked pain, flare, and secondary hyperalgesia in an experimental human model of trigeminal sensitization. Pain.

[22] Cumberbatch M.J., Hill R.G., Hargreaves R.J. (1997). Rizatriptan has central antinociceptive effects against durally evoked responses. Eur. J. Pharmacol..

[23] Akerman S., Holland P.R., Lasalandra M.P., Goadsby P.J. (2013). Endocannabinoids in the brainstem modulate dural trigeminovascular nociceptive traffic via CB1 and “triptan” receptors: Implications in migraine. J. Neurosci..

[24] Gazerani P., Au S., Dong X., Kumar U., Arendt-Nielsen L., Cairns B.E. (2010). Botulinum neurotoxin type A (BontA) decreases the mechanical sensitivity of nociceptors and inhibits neurogenic vasodilation in a craniofacial muscle targeted for migraine prophylaxis. Pain.

[25] Da Silva L.B., Kulas D., Karshenas A., Cairns B.E., Bach F.W., Arendt-Nielsen L., Gazerani P. (2014). Time course analysis of the effects of botulinum neurotoxin type A on pain and vasomotor responses evoked by glutamate injection into human temporalis muscles. Toxins.

[26] Solomon S. (2011). Botulinum toxin for the treatment of chronic migraine: The placebo effect. Headache.

[27] Colloca L., Tinazzi M., Recchia S., Le Pera D., Fiaschi A., Benedetti F., Valeriani M. (2008). Learning potentiates neurophysiological and behavioral placebo analgesic responses. Pain.

[28] De Tommaso M., Brighina F., Fierro B., Francesco V.D., Santostasi R., Sciruicchio V., Vecchio E., Serpino C., Lamberti P., Livrea P. (2010). Effects of high-frequency repetitive transcranial magnetic stimulation of primary motor cortex on laser-evoked potentials in migraine. J. Headache Pain.

[29] Garcia-Larrea L., Frot M., Valeriani M. (2003). Brain generators of laser-evoked potentials: From dipoles to functional significance. Neurophysiol. Clin..

[30] De Tommaso M., Guido M., Libro G., Losito L., Sciruicchio V., Monetti C., Puca F. (2002). Abnormal brain processing of cutaneous pain in migraine patients during the attack. Neurosci. Lett..

[31] Valeriani M., de Tommaso M., Restuccia D., Le Pera D., Guido M., Iannetti G.D., Libro G., Truini A., Di Trapani G., Puca F. (2003). Reduced habituation to experimental pain in migraine patients: A CO_2_ laser evoked potential study. Pain.

[32] De Tommaso M., Lo Sito L., di Fruscolo O., Sardaro M., Pia Prudenzano M., Lamberti P., Livrea P. (2005). Lack of habituation of nociceptive evoked responses and pain sensitivity during migraine attack. Clin. Neurophysiol..

[33] Coppola G., di Lorenzo C., Schoenen J., Pierelli F. (2013). Habituation and sensitization in primary headaches. J. Headache Pain..

[34] Cooke L., Eliasziw M., Becker W.J. (2007). Cutaneous allodynia in transformed migraine patients. Headache.

[35] Burstein R., Jakubowski M., Garcia-Nicas E., Kainz V., Bajwa Z., Hargreaves R., Becerra L., Borsook D. (2010). Thalamic sensitization transforms localized pain into widespread allodynia. Ann. Neurol..

[36] Truini A., Panuccio G., Galeotti F., Maluccio M.R., Sartucci F., Avoli M., Cruccu G. (2010). Laser-evoked potentials as a tool for assessing the efficacy of antinociceptive drugs. Eur. J. Pain.

[37] Negro A., Curto M., Lionetto L., Crialesi D., Martelletti P. (2015). OnabotulinumtoxinA 155 U in medication overuse headache: A two years prospective study. Springerplus.

[38] Headache Classification Committee of the International Headache Society (IHS) (2013). The International Classification of Headache Disorders, 3rd edition (beta version). Cephalalgia.

[39] Ashkenazi A., Silberstein S., Jakubowski M., Burstein R. (2007). Improved identification of allodynic migraine patients using a questionnaire. Cephalalgia.

[40] Sarchielli P., Granella F., Prudenzano M.P., Pini L.A., Guidetti V., Bono G., Pinessi L., Alessandri M., Antonaci F., Fanciullacci M. (2012). Italian guidelines for primary headaches: 2012 revised version. J. Headache Pain.

[41] Martelletti P., Katsarava Z., Lampl C., Magis D., Bendtsen L., Negro A., Russell M.B., Mitsikostas D.D., Jensen R.H. (2014). Refractory chronic migraine: A consensus statement on clinical definition from the European Headache Federation. J. Headache Pain.

[42] D’Amico D., Mosconi P., Genco S., Usai S., Prudenzano A.M., Grazzi L., Leone M., Puca F.M., Bussone G. (2001). The Migraine Disability Assessment (MIDAS) questionnaire: Translation and reliability of the Italian version. Cephalalgia.

[43] Langermark M., Olesen J. (1987). Pericranial tenderness in tension headache. A blind, controlled study. Cephalalgia.

[44] De Tommaso M., Sardaro M., Serpino C., Vecchio E., Franco G., Sardaro M., Delussi M., Livrea P. (2009). Fibromyalgia comorbidity in primary headaches. Cephalalgia.

[45] De Tommaso M., Libro G., Guido M., Losito L., Lamberti P., Livrea P. (2005). Habituation of single CO_2_ laser-evoked responses during interictal phase of migraine. J. Headache Pain.

[46] De Tommaso M., Sciruicchio V., Ricci K., Montemurno A., Gentile F., Vecchio E., Barbaro M.G., Simeoni M., Goffredo M., Livrea P. (2016). Laser-evoked potential habituation and central sensitization symptoms in childhood migraine. Cephalalgia.

